# Effect of screening by clinical breast examination on breast cancer incidence and mortality after 20 years: prospective, cluster randomised controlled trial in Mumbai

**DOI:** 10.1136/bmj.n256

**Published:** 2021-02-24

**Authors:** Indraneel Mittra, Gauravi A Mishra, Rajesh P Dikshit, Subhadra Gupta, Vasundhara Y Kulkarni, Heena Kauser A Shaikh, Surendra S Shastri, Rohini Hawaldar, Sudeep Gupta, C S Pramesh, Rajendra A Badwe

**Affiliations:** 1 Department of Surgical Oncology, Tata Memorial Centre, Homi Bhabha National Institute, Dr Ernest Borges Road, Parel, Mumbai 400 012, Maharashtra, India; 2 Centre for Cancer Epidemiology, Department of Preventive Oncology, Tata Memorial Centre, Homi Bhabha National Institute, Parel, Mumbai, India; 3 Centre for Cancer Epidemiology, Tata Memorial Centre, Advanced Centre for Treatment, Research and Education in Cancer, Homi Bhabha National Institute, Kharghar, Mumbai, India; 4 Department of Health Disparities Research, Division of Cancer Prevention and Population Sciences, University of Texas M D Anderson Cancer Center, Houston, TX, USA; 5 Research Administration Council, Tata Memorial Centre, Homi Bhabha National Institute, Parel, Mumbai, India; 6 Department of Medical Oncology, Tata Memorial Centre, Homi Bhabha National Institute, Parel, Mumbai, India

## Abstract

**Objective:**

To test the efficacy of screening by clinical breast examination in downstaging breast cancer at diagnosis and in reducing mortality from the disease, when compared with no screening.

**Design:**

Prospective, cluster randomised controlled trial.

**Setting:**

20 geographically distinct clusters located in Mumbai, India, randomly allocated to 10 screening and 10 control clusters; total trial duration was 20 years (recruitment began in May 1998; database locked in March 2019 for analysis).

**Participants:**

151 538 women aged 35-64 with no history of breast cancer.

**Interventions:**

Women in the screening arm (n=75 360) received four screening rounds of clinical breast examination (conducted by trained female primary health workers) and cancer awareness every two years, followed by five rounds of active surveillance every two years. Women in the control arm (n=76 178) received one round of cancer awareness followed by eight rounds of active surveillance every two years.

**Main outcome measures:**

Downstaging of breast cancer at diagnosis and reduction in mortality from breast cancer.

**Results:**

Breast cancer was detected at an earlier age in the screening group than in the control group (age 55.18 (standard deviation 9.10) *v* 56.50 (9.10); P=0.01), with a significant reduction in the proportion of women with stage III or IV disease (37% (n=220) *v* 47% (n=271), P=0.001). A non-significant 15% reduction in breast cancer mortality was observed in the screening arm versus control arm in the overall study population (age 35-64; 20.82 deaths per 100 000 person years (95% confidence interval 18.25 to 23.97) *v* 24.62 (21.71 to 28.04); rate ratio 0.85 (95% confidence interval 0.71 to 1.01); P=0.07). However, a post hoc subset analysis showed nearly 30% relative reduction in breast cancer mortality in women aged 50 and older (24.62 (20.62 to 29.76) *v* 34.68 (27.54 to 44.37); 0.71 (0.54 to 0.94); P=0.02), but no significant reduction in women younger than 50 (19.53 (17.24 to 22.29) *v* 21.03 (18.97 to 23.44); 0.93 (0.79 to 1.09); P=0.37). A 5% reduction in all cause mortality was seen in the screening arm versus the control arm, but it was not statistically significant (rate ratio 0.95 (95% confidence interval 0.81 to 1.10); P=0.49).

**Conclusions:**

These results indicate that clinical breast examination conducted every two years by primary health workers significantly downstaged breast cancer at diagnosis and led to a non-significant 15% reduction in breast cancer mortality overall (but a significant reduction of nearly 30%in mortality in women aged ≥50). No significant reduction in mortality was seen in women younger than 50 years. Clinical breast examination should be considered for breast cancer screening in low and middle income countries.

**Trial registration:**

Clinical Trials Registry of India CTRI/2010/091/001205; ClinicalTrials.gov NCT00632047.

## Introduction

The incidence of breast cancer is rising in all countries of the world,^1^ but particularly so in low and middle income countries.^2^ For example, in Mumbai, India, the incidence of breast cancer has risen by nearly 40% between 1992 and 2016,^3^ and breast cancer is the leading cause of death from cancer in women in most states of India.^4^ Breast cancers in low and middle income countries are frequently detected in advanced stages, and consequently, more than half the global deaths from breast cancer occur in these countries.^5^ While mammography is the established screening tool in developed countries, the screening modality that is appropriate for India and other low and middle income countries remains undetermined.^6^ ^7^ Breast self-examination might not be useful as a general strategy,^8^ ^9^ largely because it is not feasible to ensure women perform it well. However, a case-control study based on data from the Canadian National Breast Screening Study showed that in a controlled setting, where the quality of breast self-examination was carefully evaluated, women who conducted the procedure benefitted well.^10^ Mammography, which is widely practiced in Western countries, might not be an appropriate approach in low and middle income countries because of its cost and complexity.^6^ ^11^ Furthermore, most women in low and middle income countries are younger than 50, and mammography is less effective in this age group.^12^ ^13^

Clinical breast examination (CBE) is an alternative screening method, and was one of the components of screening in two important randomised trials.^14^ ^15^ The Health Insurance Plan Study was conducted in greater New York, USA, in the 1960s during which 62 000 women aged 40-64 were randomised to receive yearly CBE plus mammography or no screening.^14^ During the 1960s, mammography was in its early stages of development, and a disproportionately large number of breast cancers were detected by CBE. An estimated two thirds of the reduction in breast cancer mortality in the Health Insurance Plan study could be attributed to CBE.^16^

To determine the relative contributions of mammography and CBE in the reduction of breast cancer mortality, the Canadian National Breast Screening Study was initiated in the early 1980s. In one of two parts of the study, women aged 50-59 were randomly allocated to receive either yearly CBE plus mammography or yearly CBE alone.^15^ The trial had the potential to determine whether mammography provided any added benefit in terms of mortality reduction in addition to that provided by CBE. After 13 years of follow-up and five rounds of screening, deaths from breast cancer in the two arms were almost identical.^15^ These results remained unchanged after 25 years of follow-up.^17^ The findings of the Health Insurance Plan Study and Canadian National Breast Screening Study provided a strong argument for a randomised trial to compare CBE with no screening,^18^ ^19^ and formed the basis for the Mumbai study.^20^ This study aimed to determine whether CBE plus provision of cancer awareness would downstage breast cancer at diagnosis and reduce mortality from the disease, compared with no screening.

## Methods

The Mumbai study had two components: screening for cervix cancer by visual inspection and screening for breast cancer by CBE. The results of the cervical cancer component have been reported, as well as details of methodology to include design, mechanisms of community outreach, recruitment and informed consent, training of primary health workers and medical social workers, sample size estimation, adherence to screening (after three rounds), and mechanism of referral and treatment.^20^ ^21^ ^22^ The above methodological aspects are summarised in this paper.

### Definition of a cluster

A cluster comprised of many closely situated dwellings in congested slum areas, defined by geographical boundaries such as railway lines, water pipelines, highways, roads, public parks, and canals. Each cluster had 9000 to 10 000 dwellings with a population of 50 000-65 000, of which about 7500 women were aged 35-64. Transfer between control and intervention clusters was unlikely because the clusters were geographically separate, and because virtually none of the participants underwent breast screening outside the trial. The standard of care in our study population was no screening.

### Randomisation method

Randomisation was by cluster, where groups rather than individuals were chosen as units of randomisation. Twenty independent clusters were numbered 1-20 and randomly allocated to screening or control groups by a draw of lots. With this procedure, 10 clusters were assigned as screening clusters and 10 as control clusters.

### Trial participants and intervention

The current study, a cluster randomised controlled trial, recruited 151 538 women aged 35-64 from 20 clusters in Mumbai. Women in the screening arm (n=75 360) received four rounds of CBE conducted by trained female primary health workers and cancer awareness information every two years, followed by five rounds of active surveillance by way of home visits every two years. Women in the control arm (n=76 178) received one round of cancer awareness followed by eight rounds of active surveillance every two years. Participants in both arms were eligible for free diagnostic evaluation and treatment at the Tata Memorial Hospital; women in both groups were provided with identical identity cards to obtain free treatment at the hospital. Recruitment started in May 1998 and was completed in April 2002. Four rounds of CBE were concluded in December 2007 and follow-up continued until May 2018. The database was locked in March 2019 for analysis. 

### Sample size considerations

We based sample size calculations primarily on expected incidence and mortality data from breast and cervical cancer over the long duration of the study. Intracluster correlation was estimated using age, education status, and religion of women in the study. The computation was done using MLWin Software. For estimation of sample size, we considered two primary outcomes—breast and cervical cancer mortality. Sample size derived was 150 000 women, which was calculated to detect 25% reduction in mortality from breast cancer with 80% power and 5% type I error, after adjusting for intracluster correlation and design effect (0.00013758 and 2.0408, respectively). With these considerations, 230 deaths from breast cancer in the control group were required for mortality analysis to be recommended. The smaller design effect observed in the study indicated that the sample size was adequate to estimate reduction in mortality with anticipated power.

### Three way data linkage

To capture information on death from any cause, the trial had a three way data linkage system. Primary collection of data was done by trained medical social workers by home visits. Data were matched with those of Mumbai Municipal Death Records^23^ and with the Mumbai Cancer Registry.^24^ More information about the linkage systems and process has been provided in the supplementary material.

### Breast cancer deaths

Breast cancer as the cause of death among women who were diagnosed with breast cancer was blindly ascertained by two independent experts. If there was a discrepancy between the two experts, the records were blindly reviewed by a third independent reviewer. Cause of death was assigned to breast cancer when at least two of the three reviewers concurred. Cause of death could not be ascertained in 40 women.

### Statistical analysis

We calculated incidence rates in both arms by taking into account the number of person years determined from the date of entry into the trial to the date of diagnosis. The number of person years for calculating mortality rates was determined from the date of entry in the trial to the date of death. Data were censored during analysis for women who had migrated or were lost to follow-up, or who had died from other causes. All deaths in both arms were included for all cause mortality estimates. We used a Poisson regression model to estimate incidence and mortality rate ratios and their 95% confidence intervals. Adjustments were made for design effect. All statistical tests were two sided, and P<0.05 was considered to be statistically significant. The data were analysed on the basis of intention to screen (all women, irrespective of compliance), and when the predefined number of events (230 deaths) were documented in the control arm. All analyses were carried out in Stata software version 12 (Stata, College Station, TX).^25^

The study underwent several protocol amendments during its long course, particularly in the initial years. The amendments were suggested by consultants or the data safety monitoring committee from time to time and were duly approved by the institutional review board. These amendments were also approved by the funding agency (US National Cancer Institute). All interpretations in the manuscript are aligned with the finally amended protocol.

### Patient and public involvement

Patients and public were not involved in setting the research question, outcome measures, design, interpretation, or writing of the results. However, involvement of local community leaders was sought during recruitment of study participants and study implementation.

## Results

**Fig 1:**
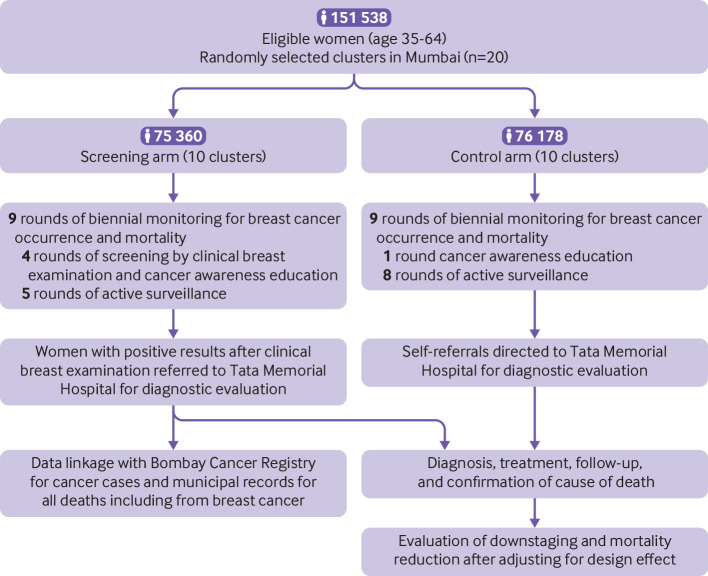
Trial flow diagram

The CONSORT flow diagram depicting the overall trial schema is presented in figure 1. Demographic and breast cancer risk factors were well balanced between the two arms indicating that randomisation was without bias (supplementary table 1). 

### Compliance, quality assurance, and breast cancer detection

The mean adherence to screening after four rounds was 67.07%, and mean adherence to hospital referral for confirmation of diagnosis was 76.21% (supplementary table 2); overall, 94.82% (n=71 456) of the participants were screened at least once. The average screen positivity rate was 1.28% in the four screening rounds (supplementary table 2). After four rounds of screening, 199 women with breast cancer were identified (supplementary table 3). Breast cancers included 114 screen detected cancers, 77 interval cancers, and eight cancers among women who did not adhere to screening in the preceding round (supplementary table 3). 

As a quality assurance measure, a random sample of 5% of women (n=10 021) was also examined by a qualified medical officer. The ĸ value for concordance was found to be 0.76, (95% confidence interval 0.72 to 0.81), indicating that the quality of CBE conducted by primary health workers met quality assurance requirements. Average adherence to rounds 5-9 of active surveillance after CBE screening was 77.57%, which was similar to the average adherence to rounds 5-9 received by the control arm (77.57% *v* 76.22%, P=0.99; supplementary tables 4 and 5). Of 641 cancers detected in the screening arm overall, 199 (31%) were detected during screening rounds 1-4 and 442 (69%) were detected during the active surveillance rounds 5-9 after CBE screening (supplementary tables 2 and 4). Adherence to treatment and to evidence based guidelines was similar in both arms (supplementary table 6); mean adherence of these women to treatment was 98.88%.

Adherence in the control arm to the first and the only round of cancer awareness was 90.88% (n=69 231). Average adherence to the subsequent eight rounds of active surveillance was 78.14% (supplementary table 5). After nine rounds of active surveillance, 655 breast cancer cases were recorded in the control arm (supplementary table 5). Progressively more breast cancers were detected in each round as the women aged. Mean adherence of these women to treatment was 97.63%.

### Age at enrolment and age at diagnosis of breast cancer

Mean age at diagnosis of breast cancer in women in the screening arm was 55.18 (standard deviation 9.10 (95% confidence interval 54.47 to 55.88)). Mean age at diagnosis in the control arm was 56.50 (9.10 (55.80 to 57.20)). This difference indicated that screening had brought forward breast cancer diagnosis by 16 months (P=0.01; table 1). At the time of recruitment, over 70% women in both the screening and control arms were younger than 50, whereas at the time of breast cancer diagnosis, this proportion was reversed with nearly 75% of women aged 50 and older in both arms (table 1). These data implied that breast cancer was diagnosed predominantly in older women, or in younger women after they reached age 50. This finding formed the basis for us to analyse the subsequent data relating to breast cancer downstaging and mortality by using age 50 as the cutoff threshold, although this threshold was not prespecified in the protocol and should be considered a post hoc analysis.

**Table 1:** Age at enrolment of all women and age at diagnosis of breast cancer

Arm	Age at enrolment (for all trial participants)							Age at diagnosis (for participants with breast cancer only)					
	Total No	No of women aged <50 (%)	No of women aged ≥50 (%)	P value	Mean age (SD (95% CI))	Difference (95% CI)		Total No	No of women aged <50 (%)	No of women aged ≥50 (%)	P value	Mean age (SD (95% CI))	Difference (95% CI)
Screening	75 177*	54 212 (72.11)	20965 (27.89)	0.06	44.84 (7.90 (44.78 to 44.90))	0.078 (−0.002 to 0.158)		640†	161 (25.16)	479 (74.84)	0.01	55.18 (9.10 (54.47 to 55.88))	1.321 (0.330 to 2.312)
Control	76 097*	54 188 (71.21)	21909 (28.79)		44.92 (8.00 (44.86 to 44.97))			655	147 (22.44)	508 (77.56)		56.50 (9.10 (55.80 to 57.20))	

SD=standard deviation.

* Information on age was not available for 183 women in the screening arm and 81women in the control arm among the total women enrolled.

† Of the 641 women with breast cancer in the screening arm, one had bilateral breast cancer, who was considered only once.

### Downstaging of breast cancer

Biennial CBE led to significant downstaging of breast cancer in all women (P=0.001; table 2), as well as in women younger than 50 (P=0.005) and in those aged 50 and older (P=0.05). Staging information was unavailable in 41 women in the screening arm and 73 women in the control arm. However, we saw no difference when comparing the survival of these women with missing information (supplementary figure 1).

**Table 2:** Staging of breast cancer at diagnosis

Age group	Randomised group	Stages I or II (No (%))	Stages III or IV (No (%))	Total No	Pearson *x* ^2^	Difference (%) in stages III+IV between screening and control arms (95% CI)
All women*	Screening arm	379 (63)	220 (37)	599	11.757 (P=0.001)	9.83 (4.208 to 15.368)
	Control arm	311 (53)	271 (47)	582		
<50†	Screening arm	271 (63)	161 (37)	432	8.034 (P=0.005)	9.77 (3.008 to 16.423)
	Control arm	206 (53)	183 (47)	389		
≥50‡	Screening arm	108 (65)	59 (35)	167	3.906 (P=0.05)	10.27 (0.094 to 20.092)
	Control arm	105 (54)	88 (46)	193		

* Staging information unavailable from 41 women in the screening arm and 73 women in the control arm.

† Staging information unavailable from six women in the screening arm and 12 women in the control arm.

‡ Staging information unavailable from 35 women in the screening arm and 61 women in the control arm.

### Breast cancer incidence and absence of overdiagnosis

At the end of screening, we found 198 women with breast cancer in the screening arm and 151 in the control arm, which translated into a crude incidence rate of 60.57 and 45.30 per 100 000 women years, respectively (rate ratio 1.34 (95% confidence interval 1.05 to 1.71); P=0.02; table 3). We saw an excess of 47 diagnoses of breast cancer in the screening arm compared with the control arm (table 3). After a median follow-up of 18 years, the screening and control arms had 640 and 655 cases of breast cancer, respectively, which translated into a crude incidence rate of 62.76 and 64.43 per 100 000 women years, respectively (0.97 (0.87 to 1.09), P=0.66; table 3). Supplementary table 7 shows that although, as expected, a higher incidence of breast cancer was seen in the screening group than in the control group up to study year 10 (that is, until the end of screening round 4), this difference reduced gradually from study year 12 onwards (starting surveillance round 1) and disappeared completely by study year 18 (surveillance round 5).

**Table 3:** Breast cancer incidence, breast cancer mortality, and all cause mortality after 20 years since commencement of study

	Screening arm					Control arm				Rate ratio (95% CI)†	P value
	Total No of women	No of diagnoses or deaths	No of person years	Crude rate per 100 000 person years (95% CI)		Total No of women	No of diagnoses or deaths	No of person years	Crude rate per 100 000 person year (95% CI)		
**Breast cancer incidence**											
Completion of active screening	75 360	198	326 891.2	60.57 (49.87 to 74.62)		76 178	151	333 346.7	45.30 (38.51 to 53.64)	1.34 (1.05 to 1.71)	0.02
Completion of 20 years of study	75 360	640	1 019 761	62.76 (57.02 to 69.35)		76 178	655	1 016 616	64.43 (60.43 to 68.90)	0.97 (0.87 to 1.09)	0.66
**Breast cancer mortality**											
All ages*	75 360	213	1 023 097	20.82 (18.25 to 23.97)		76 178	251	1 019 500	24.62 (21.71 to 28.04)	0.85 (0.71 to 1.01)	0.07
Age <50	54 212	149	763 141.8	19.53 (17.24 to 22.29)		54 188	158	751 367.0	21.03 (18.97 to 23.44)	0.93 (0.79 to 1.09)	0.37
Age ≥50	20 965	64	259 955.2	24.62 (20.62 to 29.76)		21 909	93	268 133.1	34.68 (27.54 to 44.37)	0.71 (0.54 to 0.94)	0.02
**All cause mortality**											
All ages*	75 360	11 261	1 023 180	1100.59 (989.98 to 1224.58)		76 178	11 853	101 9831	1162.25 (1037.16 to 1303.45)	0.95 (0.81 to 1.10)	0.49
Age <50	54 212	4450	763 177.7	583.09 (539.66 to 629.69)		54 188	4708	751 508.2	626.47 (572.73 to 684.32)	0.931 (0.829 to 1.045)	0.23
Age ≥50	20 965	6811	260 001.8	2619.6 (2456.3 to 2796.9)		21 909	7145	268 323.2	2662.8 (2498.2 to 2835.8)	0.984 (0.902 to 1.073)	0.71

* Information on age not available for 183 women in the screening arm and 81 women in the control arm among study participants of all ages.

† Rate ratio calculated by Poisson regression model after adjusting for cluster design.

### Breast cancer mortality

**Fig 2:**
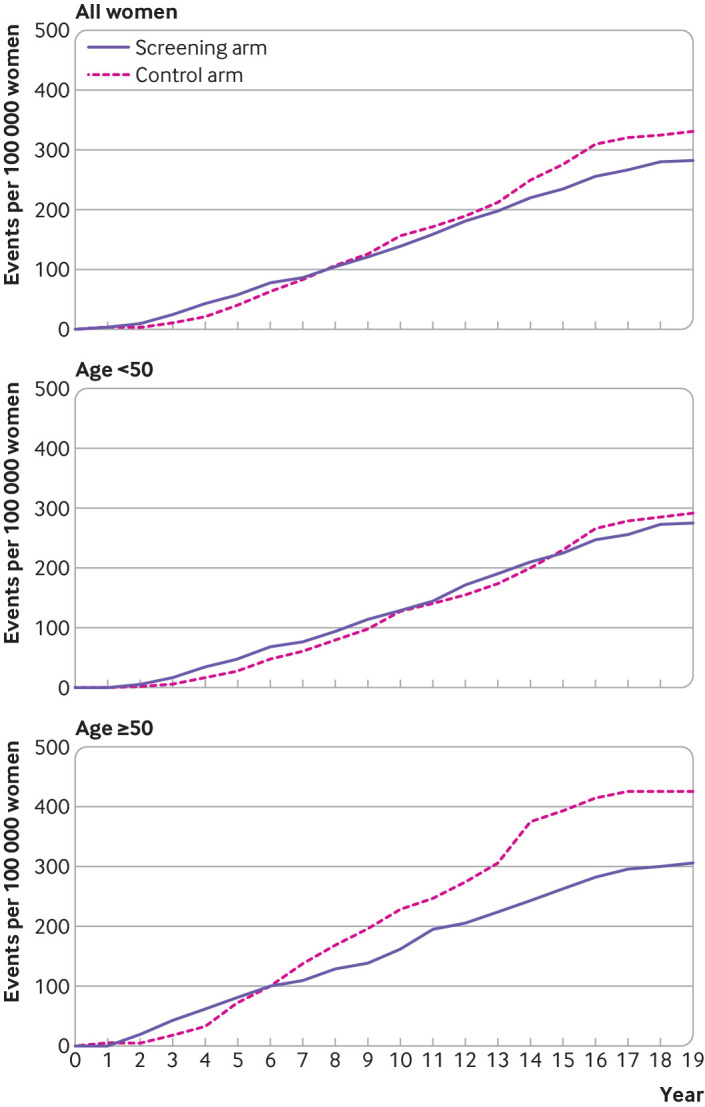
Cumulative breast cancer mortality during 20 years of study

We recorded 213 breast cancer deaths in the screening arm and 251 deaths in the control arm (rate ratio 0.85 (95% confidence interval 0.71 to 1.01), P=0.07; table 3). Thus, overall, a 15% non-significant reduction in mortality was seen when women of all ages were considered. Among women younger than 50, 149 breast cancer deaths were recorded in the screening arm and 158 deaths in the control arm (0.93 (0.79 to 1.09), P=0.37). Among women aged 50 and older, 64 breast cancer deaths were recorded in the screening arm and 93 deaths in the control arm (0.71 (0.54 to 0.94), P=0.02; table 3). This subset analysis based on the age 50 threshold was not stipulated in the protocol and was a post hoc analysis. The cumulative breast cancer mortality in the screening and control arms over 20 years is shown in figure 2. An excess of breast cancer deaths in the screened population was seen in both age subgroups (age <50 and ≥50) in the early years after randomisation (fig 2), which lasted for about 14 years in women younger than 50 and about six years in those aged 50 and older.

When breast cancer mortality data were analysed on the basis of attendance to the number of CBE screening rounds, we found that even women younger than 50 who attended all four rounds of screening benefitted significantly in terms of mortality reduction (rate ratio 0.66 (95% confidence interval 0.53 to 0.83), P<0.001). But this benefit did not exist if these women attended only three rounds (0.88 (0.60 to 1.27), P=0.48). Women aged 50 and older, however, benefitted from attending both three as well as four rounds of screening (attendance to all four rounds (0.64 (0.45 to 0.93), P=0.02); attendance to three rounds (0.66 (0.44 to 1.00), P=0.05); supplementary table 8).

### All cause mortality

When we considered all cause mortality during the 20 year period, we saw a non-significant reduction of 5% in the screening arm. All cause mortality rates were 1100.59 and 1162.25 per 100 000 women years in the screened and controls arms, respectively (rate ratio 0.95 (95% confidence interval 0.81 to 1.10); P=0.49). The subdivision of all cause mortality by age (<50 and ≥50) is also represented (table 3). Breast cancer deaths comprise less than 3% of deaths from all causes in women in India; and hence a reduction in all cause mortality was not expected. The cumulative all cause mortality in the screening and control arms over 20 years is shown in supplementary figure 2.

## Discussion

### Statement of principal findings

We report here results of our randomised trial that compared CBE screening with no screening. We showed that biennial CBE performed by trained female primary health workers significantly advanced breast cancer diagnosis by 16 months, and also downstaged the disease with fewer stage III or IV cancers in screened women. Overall, CBE led to a non-significant 15% reduction in breast cancer mortality; however, a significant reduction of nearly 30%was observed in women aged 50 and older. In women younger than 50, despite successful downstaging, no mortality reduction was observed. Lack of mortality reduction in younger women is consistent with data reported in some mammography trials,^16^ and could point to undetermined biological factors.

Participant attendance to the number of screening rounds also appeared to be important in breast cancer mortality reduction for women younger than 50. We found a 34% mortality reduction in this age group if the women attended all four rounds of screening (P<0.001). This benefit, however, disappeared if they attended only three rounds (mortality reduction 13%, P=0.48). For women aged 50 and older, however, we observed mortality reduction after attendance to three and four rounds of screening (34%, P=0.05 and 36%, P=0.02, respectively; supplementary table 8).

### Strengths and weaknesses in relation to other studies

Two other randomised trials have compared CBE screening with no screening.^26^ ^27^ A cluster randomised controlled trial was initiated in Kerala, India, in 2006 where three rounds of CBE every three years was planned to evaluate whether CBE can reduce incidence of advanced breast cancers and mortality from the disease.^26^ Early results have shown a higher proportion of early stage breast cancers in the intervention arm than in the control arm.^26^ Another trial comparing CBE screening with no screening in the Philippines could not be satisfactorily concluded because of unacceptably low levels of adherence,^27^ possibly because of external investigators not fully anticipating cultural and psychosocial barriers.

In our study, an excess mortality from breast cancer was seen in the screening arm during the first few years of screening for the total study population as well as when stratified by age groups. Such an excess mortality was also seen in the cervical cancer component of this trial.^28^ A meta-analysis of seven breast cancer screening trials^29^ suggested an excess breast cancer mortality up to the fifth year of screening in women younger than 50 and in the first year in older women. This excess was, however, not apparent in a combined analysis of Swedish trials.^30^ The possible finding of early excess cancer mortality needs exploring. The theory of biological predeterminism (pre-existing micrometastases before diagnosis and surgery) fails to explain this excess mortality but could point towards an impact of events at the time of diagnosis and surgery on mortality.^31^

### Strengths and weaknesses of this study

One crucial element of our study that led to its successful completion was that it was entirely indigenous. The trial was conceived, designed and implemented by a team based in Mumbai and had full understanding of the psychosocial, geopolitical, and geographical ground realities that influence the conduct of complex, public health randomised trials in low and middle income countries. Our study was conducted in slum areas largely inhabited by socioeconomically disadvantaged women who often moved residence requiring our medical social workers to trace their new abodes, sometimes in far flung parts of the city. Owing to our medical social workers making innumerable home visits to a population that was often mobile, we were able to achieve a satisfactory compliance at all levels of screening. The quality of CBE performed by our primary health workers was also of high standard, which was confirmed by comparing the screening findings with a specialist breast clinician. We were also able to capture death records of a high proportion of cases because of the three way data linkage system. Finally, our study included near perfect randomisation for a cluster randomised controlled trial; all demographic and breast risk factors were equally distributed in the screening and control arms. Provision of timely treatment could have helped to improve quality of life in screened women by preventing advanced stage disease, including local recurrence.

Our study also had some limitations. Cancer staging data were unavailable from 41 women in the screening arm and 73 women in the control arm. This limitation probably did not affect the study results because the survival curves of patients with missing staging information were similar in the screening and control arms (supplementary figure 1). However, a sensitivity analysis of patients with missing staging information, in which all 41 women from the screening arm were assigned cancer stages III or IV and all 73 women from the control arm were assigned to cancer stages I or II, led to loss of statistical significance in the downstaging effect of screening. Another study limitation was that cause of death information was not available through death certificates and the available documents for some women. To overcome this limitation, three independent experts reviewed the records of all women with breast cancer who had died. Breast cancer was assigned as a cause of death only when at least two reviewers concurred (213 (83%) of 258 in the screening arm and 251 (90%) of 278 in the control arm).

Our blinded review process for assigning cause of death was based on similar mechanisms used in other screening trials.^32^ ^33^ However, the possibility of some residual uncertainty cannot be excluded; some degree of variability is inevitable in screening trials when death certificates are often modestly accurate and medical records often incomplete.

We did not observe a significant reduction in all cause mortality. But because breast cancer deaths comprise less than 3% of all deaths in women in India, we did not expect a reduction in all cause mortality in our study.

### Meaning of the study—possible explanations and implications for clinicians and policymakers

Our study validates CBE as an alternative modality of breast screening. It demonstrates that CBE screening is effective in reducing breast cancer mortality in Indian women aged 50 and older without any overdiagnosis. In our trial, we were able to use a vertical programme with dedicated staff that was centrally controlled. Furthermore, women in India and in many other low and middle income countries are relatively lean and have smaller breasts than women in Western countries. The health workers who screened women with CBE in this trial had passed 10th grade education and could be trained to perform CBE within a minimal training period (about four weeks). We believe that CBE screening by primary health workers is replicable in the general population, and CBE has already been implemented in other parts of India as pilot schemes. Our study suggests that implementation of population screening by CBE in low and middle income countries is feasible, provided that adequate training of screening providers, careful monitoring, and quality of performance are assured.

Whether the use of CBE in low and middle income countries at the community level can lead to a reduction in breast cancer mortality is still unknown. Its success can only be ascertained several years after CBE has been implemented as public health programme.

**What is already known on this topic**

Breast cancer screening by mammography reduces mortality in women aged 50 and older, but its effectiveness in women younger than 50 is questionable
Breast self-examination has not been proven to be an effective method for early detection of breast cancer
Whether screening by clinical breast examination can reduce mortality from breast cancer is not known

**What this study adds**

In a 20 year study, clinical breast examination conducted by trained female health workers in Mumbai led to a downstaging of breast cancer at diagnosis and reduced mortality from the disease by nearly 30% in women aged 50 and older, but with no mortality reduction seen in women younger than 50
A 5% reduction in all cause mortality was seen in the screening arm compared with the control arm, but was not statistically significant
Clinical breast examination should be considered for breast cancer screening in low and middle income countries

## Web extra

Extra material supplied by authors

Web appendix: Supplementary material

## Notes

Contributors: IM was responsible for the conceptualisation, study design, intellectual content, community organisation, supervision of community activities, data collection, data analysis, statistical analysis, data interpretation, literature search, writing, editing, and review of the manuscript. GAM was responsible for the community organisation, monitoring and supervision of community activities, data collection, data analysis, statistical analysis, literature search, manuscript editing, and review of the manuscript. RPD was responsible for the data analysis, statistical analysis, manuscript editing, and review of the manuscript. SG was responsible for the data analysis, statistical analysis, and review of the manuscript. VYK and HKAS were responsible for the monitoring of the field work, data collection, and review of the manuscript. SSS was responsible for the community organisation, overall supervision of the field work, monitoring of the study, data collection, data analysis, and review of the manuscript. RH was responsible for data analysis, statistical analysis, manuscript editing, and review of the manuscript. SG and CSP were responsible for data analysis, manuscript editing, and review of the manuscript. RAB was responsible for concepts, study design, intellectual content, overall supervision of the field work, monitoring of the study, data collection, data analysis, statistical analysis, literature search, manuscript preparation, manuscript editing, and review of the manuscript. IM and RAB are the study guarantors. The corresponding author attests that all listed authors meet authorship criteria and that no others meeting the criteria have been omitted.

Ethical approval: The study was approved by the institutional ethics committees of the Tata Memorial Centre. In addition to the internal data safety and monitoring committee of Tata Memorial Centre, an independent data safety and monitoring board comprising international experts reviewed the trial annually.

Data sharing: The data generated by our research and those used in this article will be made available, wherever legally and ethically required.

IM and RAB confirm that the manuscript is an honest, accurate, and transparent account of the study being reported; no important aspects of the study have been omitted; and any discrepancies from the study as originally planned have been explained.

Dissemination to participants and related patient and public communities: Study results will be disseminated through scientific publications, presentations at scientific meetings, press release, social media platforms, articles in lay press, and patient and public engagement events organised by our centre.

Provenance and peer review: Not commissioned; externally peer reviewed.
