# Supplementary material for: Effect of screening by clinical breast examination on breast cancer incidence and mortality after 20 years: prospective, cluster randomised controlled trial in Mumbai
Source: BMJ. 2021 Feb 24;372:n256. doi: 10.1136/bmj.n256 (PMC7903383; doi:10.1136/bmj.n256)
Supplement: Supplementary file 1 — Web appendix: Supplementary material [file miti060253.ww.pdf]

Supplementary Table 1:

Distribution of participants by demographic and breast cancer risk factors

Variables		Screening arm N=75360 (%)	Control arm N=76178(%)
**Age groups**	35-39	22718 (30.22)	22832 (30.00)
	40-44	17311 (23.03)	17245 (22.66)
	45-49	14183 (18.86)	14111 (18.54)
	50-54	9422 (12.53)	9564 (12.57)
	55-59	6049 (08.05)	6127 (08.05)
	60-64	5494 (07.31)	6218 (08.17)
**Mean age (SD)**		44.84 (07.86)	44.92 (08.01)
**Median (IQR)**		43 (Q1 38, Q3 50, IQR 12)	43 (Q1 38, Q3 50, IQR 12)
**Marital status**	Unmarried	295 (00.40)	454 (00.60)
	Married	61584 (82.60)	62048 (82.62)
	Widowed	12289 (16.48)	12301 (16.38)
	Divorced	393 (00.53)	302 (00.40)
**Menstrual status**	Premenopausal	40361 (53.78)	40913 (53.85)
	Postmenopausal	32845 (43.77)	33456 (44.04)
	Perimenopausal	1842 (02.45)	1601 (02.11)
**Mean age at menarche (S.D)**		13.79 (01.26)	13.85 (01.24)
**Mean age at menopause (S.D)**		43.36 (04.96)	43.62 (04.87)
**Mean age at marriage (S.D)**		17.99 (03.89)	17.85 (03.82)
**Mean age at first child birth (S.D)**		20.82 (03.70)	20.74 (03.61)
**Average number of children (S.D)**		3.58 (01.61)	3.56 (01.61)
**Breast feeding (%)**	Yes	70002(98.85)	70389 (98.64)
	N o	811 (01.15)	971 (01.36)
**Family history of breast cancer (%)**	Yes	517 (00.70)	504 (00.68)
	N o	73312 (99.30)	74050 (99.32)
**Previous consultation for breast-related complaints (%)**	Yes	882 (01.21)	785 (01.06)
	N o	72105 (98.79)	73142 (98.94)

Supplementary Table 2:

Compliance to screening, referral and breast cancer cases detected till round 4 in screening arm

Screening Round	Eligible Women	Women Examined	Compliance to Screening (%)	Screen +ve cases N (%)	Compliance of screen +ve cases to referral N (%)	Breast Cancers Confirmed
**One**	75360	56985	75.62	350 (0.61)	239 (68.29)	32
**Two**	74922	49012	65.42	552 (1.13)	393 (71.20)	55
**Three**	74211	47133	63.51	638 (1.35)	503 (78.84)	42
**Four**	73241	46554	63.52	1024 (2.20)	819 (79.98)	70
**Total &/ or Mean**	**74433**	**49921**	**67.07**	**1.28**	**76.21**	**199**

*****One participant was considered twice because of bilateral breast cancer

Supplementary Table 3:

Breast cancers detected during 4 rounds of screening

**Round One**	Screen detected	32
	Interval cancers	26
	Non- Compliers	04
**Round Two**	Screen detected	25
	Interval cancers	14
	Non- Compliers	01
**Round Three**	Screen detected	27
	Interval cancers	37
	Non- Compliers	03
**Round Four**	Screen detected	30
**Total**		**199**

*One participant was considered twice because of bilateral breast cancer

Supplementary Table 4:

Compliance to active surveillance and breast cancer cases recorded during rounds 5 – 9 in screening arm

Round	Eligible Women	Compliance to Surveillance (%)	Breast Cancers
Five	71886	57549 (80.06)	74
Six	70455	55310 (78.50)	75
Seven	69006	53732 (77.87)	74
Eight	67437	51951 (77.04)	67
Nine	64099	47705 (74.42)	152
Total breast cancers			442

Supplementary Table 5:

Compliance to active surveillance and breast cancer cases recorded from round 1- 9 in control arm

Round	Eligible women	Compliance to surveillance (%)	Breast cancers
**One**	76178	69227 (90.88)	2
**Two**	75654	62763 (82.96)	38
**Three**	74842	59541 (79.56)	51
**Four**	73768	59303 (80.39)	60
**Sub-total after 4 rounds of active surveillance**			**151**
**Five**	72341	56689 (78.36)	74
**Six**	70914	54576 (76.96)	87
**Seven**	69381	53158 (76.62)	83
**Eight**	67784	51427 (75.87)	81
**Nine**	64325	47143 (73.29)	179
**Sub-total in rounds 5-9 of active surveillance**			**504**
**Total breast cancers**			**655**

Supplementary Table 6

Women availing breast cancer treatment at TMH and Other Hospitals

Treatment Taken	Screening Arm (%)	Control Arm (%)	Pearson x^2 P-value
TMH as well as other hospitals	413 (64.53)	399 (60.91)	x^2 =1.717<br>p=0.190
Other hospitals only	227 (35.47)	255 (38.93)	
No information	-	1(0.15)	
**Total**	**640**	**655**	

Supplementary Table 7:

Cumulative breast cancer cases in Screening and Control Arm during successive rounds

**Screening arm**		**Control arm**	
Screening Round 1	32	Active Surveillance Round 1	2
Screening Round 2	87	Active Surveillance Round 2	40
Screening Round 3	129	Active Surveillance Round 3	91
Screening Round 4	199	Active Surveillance Round 4	151
Active Surveillance Round 1	273	Active Surveillance Round 5	225
Active Surveillance Round 2	348	Active Surveillance Round 6	312
Active Surveillance Round 3	422	Active Surveillance Round 7	395
Active Surveillance Round 4	489	Active Surveillance Round 8	476
Active Surveillance Round 5	641	Active Surveillance Round 9	655

Supplementary Table 8:

Breast cancer mortality on the basis of attendance to number of screening rounds (3 or 4)

	Screening arm				Control arm				Rate ratio (95% CI)	P-Value
	No. of women	No. of breast cancer deaths	Person years	**Crude rate (95% CI)	No. of women	No. of breast cancer deaths	Person years	**Crude rate (95% CI)		
**Breast cancer mortality: Screened in all 4 screening rounds**										
**Age***	24984	62	394239.1	15.726 (13.57 to 18.43)	76178	251	1019500	24.620 (21.71 to 28.04)	0.639 (0.526 – 0 776)	<0.001
**< 50**	19296	43	309187.8	13.907 (11.41 to 17.16)	54188	158	751367	21.028 (18.97 to 23.44)	0. 661 (0.527 – 0.830)	<0.001
**≥ 50**	5688	19	85051.27	22.339 (16.52 to 30.46)	21909	93	268133.1	34.684 (27.54 to 44.37)	0.644 (0.445 – 0.931)	0.02
	**Screening arm**				**Control arm**				**Rate ratio (95% CI)**	**P-Value**
	**No. of women**	**No. of breast cancer deaths**	**Person years**	****Crude rate (95% CI)**	**No. of women**	**No. of breast cancer deaths**	**Person years**	****Crude rate (95% CI)**		
**Breast cancer mortality: Screened in 3 screening rounds**										
**Age***	19825	57	291331.5	19.565 (14.69 to 26.76)	76178	251	1019500	24.620 (21.71 to 28.04)	0.795 (0.578 – 1.092)	0.16
**< 50**	14482	40	217467.3	18.394	54188	158	751367	21.028	0.875	0.48

				(12.88 to 27.67)				(18.97 to 23.44)	(0.603 – 1.269)	
**≥ 50**	5342	17	73864.24	23.015 (16.14 to 33.39)	21909	93	268133.1	34.684 (27.54 to 44.37)	0.664 (0.439 – 1.002)	0.05

*Information on age was not available for 183 women in screening arm and 81 women in control arm among the total women enrolled

**Crude rate per 100000 person years. #Rate Ratio = Rate Ratio calculated by ‘Poisson regression model ‘after adjusting for ‘cluster design’.

Supplementary Figure 1: Survival of women in whom staging information was unavailable; 41 cases in screening and 73 cases in control arms.

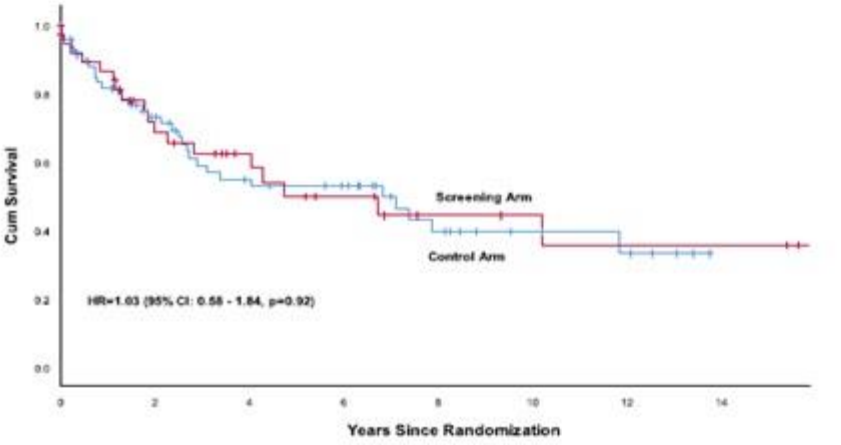

No. of Patients								
Screening Arm	40	22	15	10	6	5	4	3
Control Arm	73	39	26	22	12	7	5	1

Supplementary Figure 2: Cumulative all-cause mortality over twenty years.

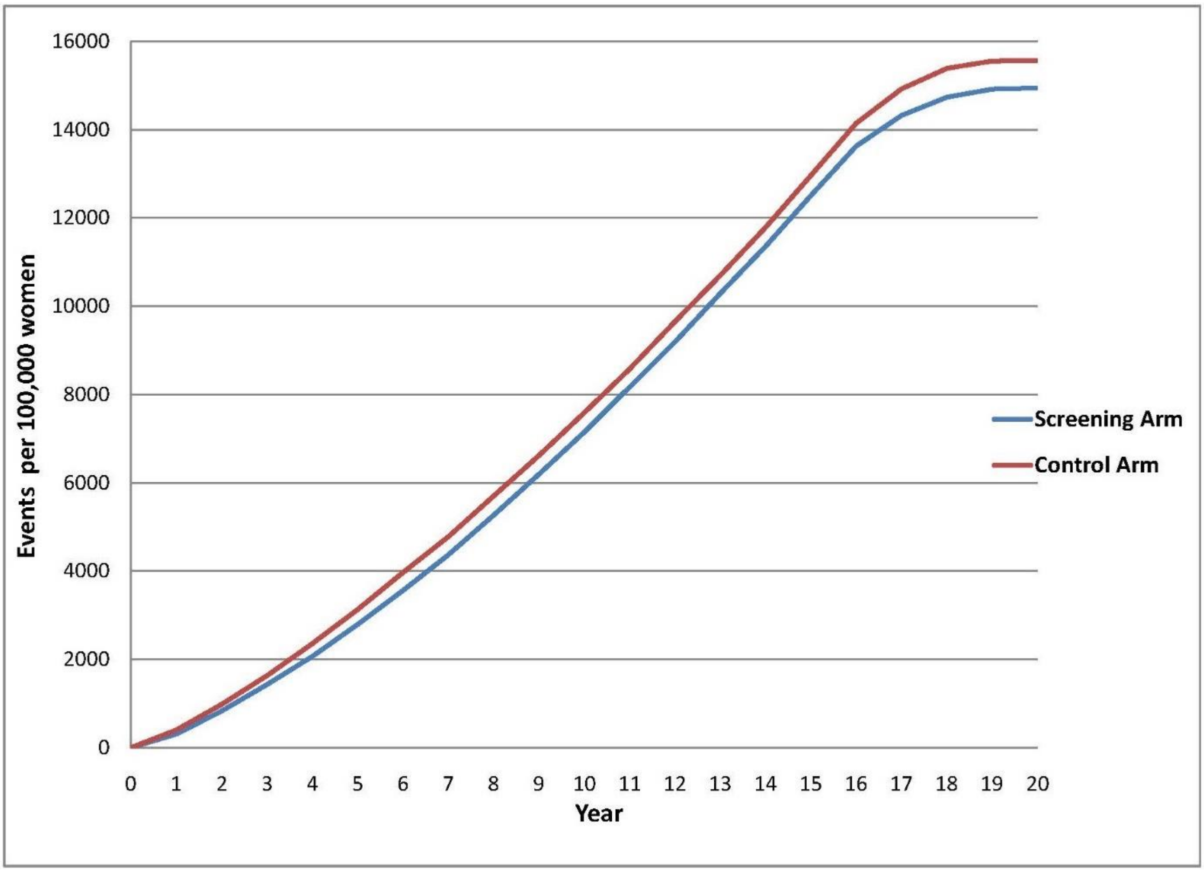

Information about the linkage systems and process:

The primary collection of follow up data was done through individual household visits by our Medical Social Workers who collected information on ~85% of incident and death cases in the study. The rest of the data were collected through linkages with Municipal Corporation of Greater Mumbai (MCGM) and Mumbai Cancer Registry.

The vital statistics division of the Municipal Corporation of Greater Mumbai (MCGM) has one of the most advanced birth and death record registries in India. Records have been validated through independent audits to be 95% complete. Records of MCGM are computerized and available within six months of an event of birth or death. The municipal death records were obtained every six months and were matched with the primary trial data mentioned above. Finally, our Medical Social Workers made individual home visits to corroborate details of women in study which were matched with the MCGM data.

The Bombay Cancer Registry, established in 1963, is the oldest population based cancer registry to be set up in India. This registry was started with inputs from the NIH and the Descriptive Epidemiology Unit of IARC. Subsequently, the registry staff were further trained at the University of Tampere, Finland under the guidance of Prof. Matti Hakama. The Registry collects cancer related information from all major hospitals and private nursing homes. The major cancer hospitals are visited daily, while other hospitals and nursing homes are visited once a week. The maximum time lag between a diagnosed case of cancer and the availability of this information at the Bombay Cancer Registry is one week. It however takes three years for the registry to collate, quality check and publish the data. A quality control survey conducted by Indian Council of Medical Research (ICMR) to assess the quality and completeness of the Bombay Cancer Registry records shows a completeness of 95.4%.

Reference:

Yeole BB. An assessment of improvement in reliability and completeness of Mumbai cancer registry data from 1964-1997. Asian Pac J Cancer Prev. 2001;2(3):225-32.
